# Hypotension after Induction of Anesthesia as a Predictor of Hypotension after Opening the Dura Mater during Emergency Craniotomy

**DOI:** 10.3390/jcm13196021

**Published:** 2024-10-09

**Authors:** Izabela Duda, Mariusz Hofman, Mikołaj Dymek, Piotr Liberski, Maciej Wojtacha, Anna Szczepańska

**Affiliations:** 1 Department of Anaesthesiology and Intensive Care, Faculty of Medical Sciences, Medical University of Silesia, 40-055 Katowice, Poland; mariusz.hofman@sum.edu.pl (M.H.); pliberski@sum.edu.pl (P.L.); aszczepanska@sum.edu.pl (A.S.); 2 Department of Neurosurgery, University Clinical Center of the Medical University of Silesia, 40-055 Katowice, Poland; mikolajdym@gmail.com (M.D.); macwojt@poczta.fm (M.W.)

**Keywords:** intraoperative hypotension, hypertension, traumatic brain injury, emergency craniotomy, acute subdural haematoma

## Abstract

**Background:** The subject of this study is intraoperative hypotension during the evacuation of acute subdural haematoma (ASH). We examined the association between the decrease in intraoperative blood pressure (BP) after the induction of anaesthesia and the decrease in BP after opening the dura mater. The second aim of this study was to assess the relationship between preoperative hypertension and the emergence of an intraoperative drop in BP. **Methods:** This was a retrospective cohort study on adult patients undergoing emergency craniotomy due to ASH. In total, 165 medical records from a 2-year period were analysed. The patients were divided into two groups: high blood pressure (HBP) (n = 89) and normal blood pressure (NBP) (n = 76). The HBP group included patients with hypertension in the preoperative period (systolic blood pressure (SBP) > 150 mmHg). The NBP group included patients with an SBP between 90 and 150 mmHg. **Results:** We observed a significant drop in blood pressure in two operational periods: after the induction of anaesthesia and after opening the dura mater. A highly relevant positive correlation was noted between the decrease in SBP after anaesthesia induction and the opening of the dura mater (*p* < 0.001). In the HBP group, after opening the dura mater, there was a 44% SBP decrease from the baseline value. **Conclusions:** The reduction in BP after the induction of anaesthesia is a predictor of a subsequent drop in BP after opening the dura mater during urgent surgery due to ASH. Patients with hypertension in the preoperative period of ASH tend to have a greater intraoperative drop in BP and worse outcomes.

## 1. Introduction

Craniocerebral injuries are a great social and economic problem. Their incidence in highly industrialised countries is estimated at about 200,000 people per year. They are one of the leading causes of death in all age groups. The incidence of deaths due to craniocerebral injuries is about 20 per 100,000 people per year. The effects of injuries often require long-term treatment and rehabilitation [1].

Among the various forms of traumatic brain injury (TBI), cases requiring immediate surgery to remove the mass effect deserve special attention. Acute subdural haematoma (ASH) occurs in 12–29% of severe TBI cases and has mortality rates ranging from 40% to 60% [2].

Patients with ASH must often be operated on urgently in order to evacuate intracranial haematomas. During the operation, a sudden drop in blood pressure may be observed, particularly after opening the dura mater [3,4,5,6]. The haemodynamic changes that result from the sudden drop in intracranial pressure (ICP) cause an inverse Cushing response and, as a result, sudden hypotension [7,8]. Patients with TBI and hypotension during emergency craniotomy tend towards worse outcomes [9]. Thus, predicting and avoiding intraoperative hypotension is an important clinical problem. The connection between systemic hypotension and worse outcomes in patients after TBI have been described in many studies [10,11,12,13,14,15,16]. However, the incidence and risk factors of intraoperative hypotension are not well diagnosed. Intraoperative hypotension occurs in 65% of patients [7]. The main risk factors are blood loss, inadequate preoperative fluid resuscitation, changes in autonomic system tension, general anaesthesia, a low Glasgow scale score, tachycardia and preoperative hypertension [4]. Hypertension is observed in 25% of patients with TBI and results from a reaction of the sympathetic nervous system [17]. Hypertension can be a signal of the effort to maintain autoregulation to optimise cerebral blood flow and cerebral perfusion pressure. Nevertheless, it may lead to cerebral oedema and an increase in ICP [18].

During the induction of anaesthesia, blood pressure often drops. This occurs in about 30% of patients. A drop in blood pressure during induction may be due to multifactorial causes, including the type and dose of anaesthetic agent used, a high American Society of Anesthesiologists (ASA) score, hypovolemia, or ventricular dysfunction. It is the result of venous dilatation, arterial dilatation, or decreased cardiac contractility, but the contributions of different haemodynamic mechanisms to hypotension remain unclear [19,20,21,22].

In our study, we examined systemic pressure during emergency craniotomy due to ASH. The aim of this study was to find the relationship between a decrease in blood pressure after the induction of anaesthesia and after opening the dura mater. We hypothesised that a decrease in blood pressure after the induction of anaesthesia may be a predictor of a decrease in blood pressure after opening the dura mater.

The second aim was to determine a relationship between the systemic pressure value in the preoperative period and observed changes during the operation.

## 2. Materials and Methods

The retrospective cohort study involved adult patients (18 years and above) who underwent emergency craniotomy due to ASH. The Ethics Committee of the Medical University of Silesia of Katowice approved the study protocol and waived the need to obtain informed consent for participation in the study from the included patients (KNW/0022/KB1/86/13). All patients’ data were obtained in accordance with the national regulations of personal data management.

The anaesthesia protocols and medical records of consecutive patients undergoing emergency craniotomy due to ASH over a 24-month period were analysed. Qualification for the procedure was performed by a neurosurgeon based on Bullock’s criteria [23].

Patients were excluded from the study in the event of systolic blood pressure (SBP) <90 mmHg after admission to the operating room (OR), undergoing vasopressor therapy, undergoing repeated surgery, and exhibiting polytrauma.

The main analysed parameter was systolic blood pressure. Patients were divided into two groups depending on the SBP value recorded after admittance to the OR. The HBP group comprised patients whose SBP exceeded 150 mmHg, and the NBP group comprised patients whose SBP was in the range of 90 to 150 mmHg.

The SBP value was recorded in four periods of the operation: BP1—the value after admittance to the OR, BP2—the lowest SBP value in the period between the induction of anaesthesia and the beginning of the operation, BP 3—the lowest SBP value in the period from the opening of the dura mater to the beginning of dural suturing, and BP 4—the SBP value at the moment of transfer from the OR.

Intraoperative blood pressure was measured using an automatic, non-invasive method with an interval of no longer than 5 min. Other quantitative and qualitative clinical parameters were also analysed. The quantitative parameters included age, Glasgow Coma Scale (GCS), intraoperative rates of diuresis and fluid therapy, haematocrit level and heart rate at admission to the OR. The qualitative parameters included sex, features of intraoperative cerebral oedema reported by a neurosurgeon based on a subjective assessment, and the receipt of pharmacological vasopressive medications (norepinephrine). In the postoperative period of staying in the hospital from the operation to discharge, the incidence of premature mortality and Glasgow Outcome Score (GOS)—for 3 postoperative months—were determined.

The statistical analysis was conducted using Statistica for Windows 9.0 statistical package. The normality of distribution was assessed using the Shapiro–Wilk test. The following characteristics for quantitative variables were calculated: mean, standard deviation, median, minimum and maximum values of the variables, and lower and upper quartiles. The analysis of SBP values, according to the examined group and measurement point, was carried out by means of repeated measures analysis of variance and Tukey’s test. The analyses of relationships between the values at the test points and the dependence on quantitative variables were conducted through the estimation of Spearman’s coefficient of linear correlations. The differences in SBP values depending on qualitative values were analysed using Student’s *t*-test. The comparison of premature mortality and the GOS scale score between groups was conducted using the chi-squared test. A value of *p* < 0.05 was considered significant, and *p* < 0.01 was considered highly significant.

## 3. Results

In the 24-month analysed period, 171 emergency craniotomies were performed due to ASH. Intraoperative protocols were available. Six protocols were excluded: one because of difficult prolonged intubation, two owing to intensive intraoperative bleeding, and three due to errors in recording data. The injury mechanisms were as follows: traffic accident (45%), fall (28%), battery (28%), and unknown reasons (16%) (patient found). In 14% of cases, the condition was recognised after alcohol consumption.

A total of 89 patients were assigned to the HBP group, and 76 patients were assigned to the NBP group. The patients in the HBP group had a faster heart rate after admittance to the OR, as well as a higher haematocrit value and smaller volume of intraoperative diuresis compared with the patients in the NBP group. The pre- and intraoperative patient variables are shown in Table 1.

**Figure 1:**
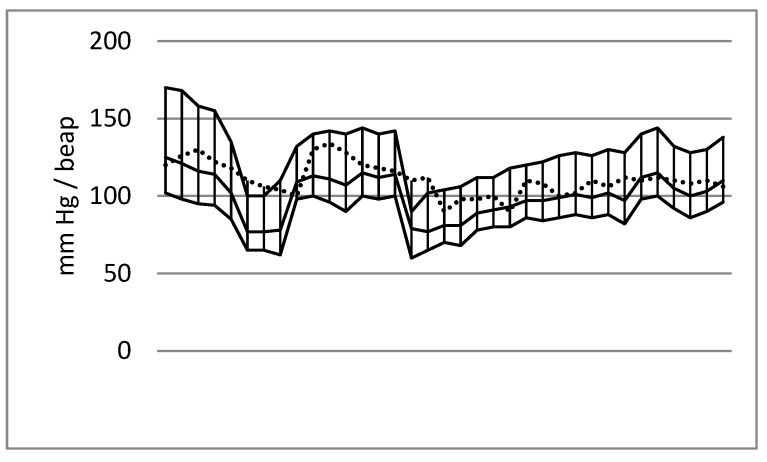
Blood pressure values (systolic blood pressure, mean blood pressure and diastolic blood pressure: line max_min) and heart rate (dashed line) during emergency craniotomy due to acute subdural haematoma. Male patient aged 39; cause of injury: fall. The first decrease in blood pressure was noted after anaesthesia induction, and a second decrease occurred after opening the dura mater.

The data analysis indicated characteristic blood pressure trends during the emergency craniotomies. A decrease in values was observed after the induction of anaesthesia and after the opening of the dura mater, whereas an increase in values was typical of the beginning of the operation and the final stage of the surgery. A diagram of the systolic, mean and diastolic arterial pressure trends is presented in Figure 1.

**Figure 2:**
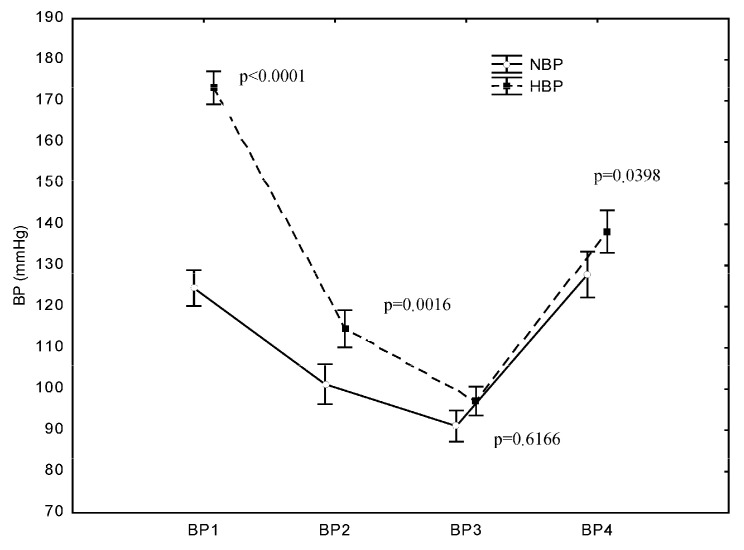
Differences in systolic blood pressure (SBP) between the HBP and NBP groups at the examined operation points. *p* represents the difference in SBP between the NBP and HBP groups at the studied measurement point: B1—before the surgery, B2—after the induction of anaesthesia, B3—after opening the dura mater, and B4—after the surgery.

In both groups, SBP dropped immediately after the induction of anaesthesia and after the opening of the dura mater and then rose after the completion of the operation. The mean SBP values at BP1 (after admittance to the OR) were 173 mmHg (±23) in the HBP group and 124 mmHg (±13) in the NBP group. At BP2 (after the induction of anaesthesia), a highly significant decrease in SBP compared with the SBP at BP1 (*p* < 0.0001) occurred in both the HBP group and NBP group. Likewise, at BP3 (after opening the dura mater), another significant decrease in SBP was recorded in both groups compared with the SBP at BP1 (*p* < 0.0001). At BP4 (after completion of the operation), a significant increase in SBP was recorded in both groups. In the NBP group, the SBP value after completion of the operation did not significantly differ from the value at BP1, i.e., it returned to the baseline value (*p* = 0.9625). In the HBP group, the increase in SBP after completion of the operation did not result in a value similar to preoperative SBP, and the post-completion SBP value was significantly lower than the value recorded at BP1 (*p* < 0.0001) (Figure 2).

**Figure 3:**
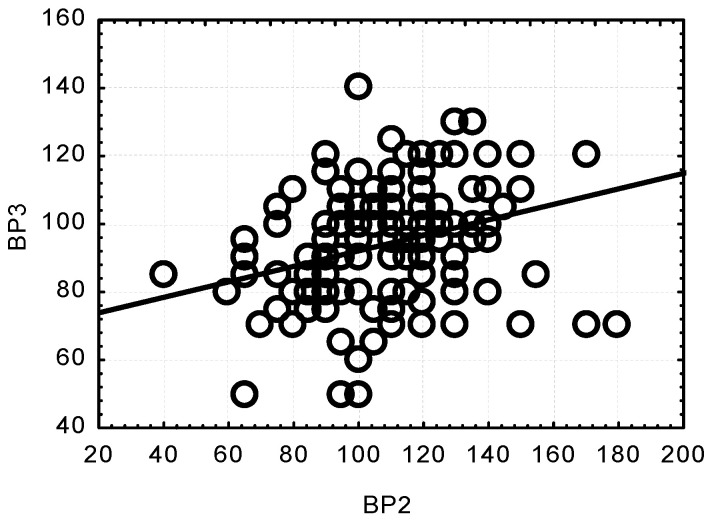
Correlation between changes in systolic blood pressure (SBP) after anaesthesia induction (BP2) and after opening the dura mater (BP3); (*p* < 0.0001; r = 0.3042).

Concurrently, significant and highly significant correlations were observed between the SBP values at the respective measurement points. All the correlations were positive. A low SBP value at one measurement point was related to a low SBP value at another point. Only the relationship between the results at BP2 and BP4 turned out to be low and insignificant, whereas there was a positive, highly significant relationship between BP2 and BP3 (*p* < 0.0001) (Figure 3).

In the HBP group, the heart rate, haematocrit at BP1, and GCS score at BP4 were highly positively correlated with the SBP value. Age at BP2 and BP1 was negatively correlated. In the NBP group, positive correlations were noted for urine output at BP2 and the GCS score at BP4. The intravenous fluid level at BP2 and HR at BP4 were negatively correlated (Table 2). Patients with intraoperative vasopressors applied were characterised by a lower SBP value at BP2 and BP3 (*p* < 0.0001). No correlation was found between patient sex, intraoperative cerebral oedema, blood transfusion, operation time, and SBP values at the examined measurement points (Table 2).

In the HBP group, a significant correlation was noted between the assessment of outcomes using the GOS scale and the value of SBP at BP1. A significant positive correlation with the value of SBP at BP3 was also noted. In the NBP group, similar correlations were not observed. Thirty-nine per cent of patients from the HBP group died after the operation (Table 3).

## 4. Discussion

This study demonstrates that a decrease in systemic pressure during emergency craniotomy may occur at two periods of the operation: after the induction of anaesthesia and after opening the dura mater. The decrease in SBP after opening the dura mater is more distinct than after the induction of anaesthesia. The strong positive correlation between SBP after the induction of anaesthesia and after opening the dura mater indicates that the drop in SBP after the induction of anaesthesia may be a predictor of a drop in BP after opening the dura mater. This fact allows the implementation of therapeutic procedures to minimise adverse haemodynamic effects after opening the dura mater. Intensive fluid resuscitation and the administration of vasopressive medications to treat hypotension after opening the dura mater may lead to acute cerebral oedema [24]. In our study, the cerebral oedema appeared sporadically and was not correlated with SBP values. The disturbance of the balance between sympathetic and parasympathetic activity is one of the theories explaining a decrease in BP after opening the dura mater [3]. Opening the dura mater leads to a sudden drop in intracranial pressure (ICP). The haemodynamic changes resulting from a drop in ICP may cause an inverse Cushing response. A decrease in ICP may lead to a decrease in BP due to the injury of central circulation regulatory areas [25]. This decrease might result from the remission of the Cushing response as a vasopressor response to ICP increases. If the Cushing response conditioned the preoperative BP, patients without preoperative hypotension, rather than patients with preoperative hypotension, could develop severe intraoperative hypotension due to larger brainstem ischaemia. In our study, the decrease in BP in patients with symptomatic hypertension was relevantly higher, but the absolute values of BP after opening the dura mater were 90 mmHg on average, regardless of the initial BP value.

Another cause of severe intraoperative hypotension after dural opening during craniectomy due to traumatic brain injury may be coagulation disorders [26].

Kinoshita et al. reported all episodes of intraoperative hypotension during emergency craniotomy after opening the dura mater [4]. However, in a study by Sharma et al., hypotension, defined as a decrease in SBP to <90 mmHg, was recorded several times during emergency craniotomy and also after the induction of anaesthesia [6]. Patients with the dysfunction of the autonomic nervous system (ANS) for various reasons, e.g., diabetes, advanced age, coronary disease, and some drug treatments, have a risk of hypotension after the induction of anaesthesia. A possible mechanism is that a homeostatic reflex in patients with ANS dysfunction is less able to compensate for the effects of anaesthesia induction on venous return, vascular tone, and myocardial contractility [19]. Autonomic dysfunction may appear after various types of brain injury [27]. One form of this dysfunction is paroxysmal sympathetic hyperactivity (PSH). PSH occurs in 8–33% of patients with TBI [28]. The main symptoms of PSH include hypertension, tachycardia, tachypnoea, and hyperpyrexia. The volume of response has a direct relationship with the severity of TBI. We observed preoperative hypertension in 54% of patients, and heart rate exceeded 100 beat/min in this group of patients. Systemic hypertension is often observed in the acute phase after head injuries [16,29,30]. All injuries, including TBI, provoke an autonomic nervous system response. The activation of the sympathetic nervous system leads to a rise in plasma catecholamine levels. There is a simple correlation between the severity of TBI and the catecholamine level [31]. In our study, we did not observe a correlation between the GCS score and the BP value before and during emergency craniotomy. However, we recorded a positive correlation between the GCS score and the BP value after the operation. This correlation may result from the stress reaction related to the recovery of consciousness after finishing anaesthesia in patients with better neurological conditions. In the present study, in the HBP group, the BP value dropped after opening the dura mater by 44% from the initial value. This drop was almost two-fold higher than the drop in the NBP group.

A decrease in BP after anaesthesia induction but before surgical stimulation is generally observed. It appears most often in the first 5–10 min after induction. This decrease is of particular concern for patients over 50 years of age with heart disease, arterial hypertension, and receiving antihypertensive drugs [19]. We also found a significant relationship between patient age and the decrease in BP both after the induction of anaesthesia and after opening the dura mater. The intraoperative decrease in BP may be the reason for the development of postoperative ischaemic stroke. Bijker et al. reported that in cases with a decrease in mean blood pressure of more than 30%, there is a high risk of postoperative ischaemic stroke. That study concerned elective non-cardiac and non-neurosurgical surgery [32]. In cases of patients receiving emergency procedures due to brain injury, intraoperative decreases in BP may considerably worsen the outcome. The correlations between BP and intravenous fluid, urine output, and HR in the intra- and postoperative period in the normotensive group might be interpreted as indicators of intraoperative fluid resuscitation. Patients with hypertension did not present any similar correlations. In the arterial hypertension group, heart rate in the preoperative period was significantly faster than in patients with normotension. This result may be a result of PSH as well as hypovolemia resulting from mannitol administration and the restriction of fluid resuscitation at the emergency stage. The administration of mannitol to emergency-stage patients with TBI may cause anterograde iatrogenic hypotension [33]. This is confirmed by a higher haematocrit value and lower intraoperative total urine output compared with patients with normotension and a strong positive correlation between haematocrit and BP after admittance to the OR. In our study, patients with preoperative arterial hypertension were characterised by a worse outcome, as assessed using the GOS. A higher BP value was recorded before the operation, and a lower value was recorded after opening the dura mater. In contrast, in preoperative normotensive patients, all correlations were positive, which means that the higher the pressure (from 90 to 150 mmHg), the better the GOS. The postoperative mortality was 43% in the preoperative hypertension group, and it was significantly higher compared to patients with normotension.

In the case of ASH, qualification for surgery is not always straightforward, especially in cases of patients in good general condition without focal neurological deficits. Servadei et al. [34] developed a protocol for selecting patients in comas for conservative treatment. The criteria for selecting patients in comas for nonoperative treatment included clinical stability or an improvement in the patient’s condition from the time of injury to the time of hospital assessment, a haematoma thickness less than 10 mm, and the displacement of midline structures of the brain at less than 5 mm on the initial computed tomography (CT), as well as monitoring intracranial pressure (ICP). Surgery was performed when the ICP exceeded 20 mmHg. According to this protocol, 15 out of 65 patients in comas were treated conservatively, and 2 patients qualified for elective surgery due to increasing ICP and the development of intracranial haematoma. Since this protocol proved effective, the authors concluded that conservative treatment can be safely used in a specific group of patients in comas with ASH. Previously, the criteria for qualification for the procedure were developed by Bulloc et al. and are still valid today, overlapping with the above [23,34].

One limitation of this study is its retrospective character, as it is based on medical documentation. Another limitation is the lack of data from the period before admittance to the OR. The medical documentation from the emergency period did not provide accurate data from the period between the injury and admittance to the OR. The admitted patients were unable to provide information to clinicians that required patient recall, such as arterial hypertension history and preoperative medication usage. Despite those limitations, this study provides a new outlook for intraoperative changes in BP during emergency craniotomy. Future studies are required to establish the treatment strategy in patients experiencing destabilisation of the sympathetic nervous system after TBI.

## 5. Conclusions

A decrease in BP after the induction of anaesthesia is a predictor of a subsequent decrease after opening the dura mater. The strong relationship between preoperative hypertension and a relevant decrease in BP during operations requires a particular therapeutic strategy to minimise intraoperative fluctuations in arterial pressure and to improve outcomes.

## Figures and Tables

**Table 1:** Preoperative and intraoperative characteristics of preoperative hypertensive patients (HBPs) and normotensive patients (NBPs).

	HBP (n = 89)	NBP (n = 76)	*p*-Value
Sex (M/F)	75/14	56/20	0.0692
Age (yr)	57.8 ± 13.7	56.8 ± 14.3	0.6541
GCS (range)	7.4 ± 4.1 (3–15)	8.7 ± 4.2 (3–15)	0.0764
Haematocrit (%)	40.2 ± 5.1	37.9 ± 4.8	0.0075 *
Surgery time (min)	108.5 ± 34.8	106.4 ± 35.1	0.6944
HR on arrival to the OR (beat/min)	100.7 ± 23.7	91.7 ± 22.8	0.0143 *
Total infusion (mL)—crystalloids	1951.1 ± 902.6	2011.1 ± 810.6	0.6559
Vasopressor use (n %)—norepinephrine	12 (13.4%)	11 (14.4%)	0.5151
Blood transfusion (n %)	8 (8.9%)	6 (7.8%)	0.5139
Total urine volume (ml)	290.8 ± 366.5	494.4 ± 475.9	0.0022 *
Intraoperative brain oedema (n %)	17 (19.1%)	12 (15.7%)	0.3638

Data are presented as mean ± SD. * significantly different. GCS = Glasgow Coma Scale; HR = heart rate; and OR = operating room.

**Table 2:** Correlation between systolic blood pressure (SBP) values at the examined measurement points and selected clinical parameters for preoperative hypertensive patients (HBPs) and normotensive patients (NBPs).

	BP 1		BP 2		BP 3		BP 4	
	HBP	NBP	HBP	NBP	HBP	NBP	HBP	NBP
Age (year)	r = 0.0266	r = −0.1349	**r = −0.2670**	r = −0.1091	**r = −0.3830**	r = −0.1738	r = 0.0160	r = −0.1115
	*p* = 0.8170	*p* = 0.2480	***p*** = 0.0180 *	*p* = 0.3510	***p*** = 0.0010 *	*p* = 0.1360	*p* = 0.8900	*p* = 0.3410
Glasgow Coma Scale	r = −0.2287	r = 0.1997	r = −0.1822	r = −0.1106	r = 0.2918	r = 0.2369	**r = 0.2635**	**r = 0.3193**
	*p* = 0.0520	*p* = 0.1080	*p* = 0.1230	*p* = 0.3770	*p* = 0.0120	*p* = 0.0550	***p*** = 0.0240 *	***p*** = 0.0090 *
HR (beap/min)	**r = 0.2155**	r = −0.0981	r = 0.1455	r = 0.0967	r = 0.0900	r = −0.0778	r = −0.0584	**r = −0.2915**
	***p*** = 0.0430 *	*p* = 0.3990	*p* = 0.1740	*p* = 0.4060	*p* = 0.4010	*p* = 0.5040	*p* = 0.5870	***p*** = 0.0110 *
Ht (%)	**r = 0.2527**	r = 0.0445	r = −0.1648	r = 0.2006	r = 0.0813	r = 0.1824	r = 0.1273	r = −0.0336
	***p*** = 0.0270 *	*p* = 0.7250	*p* = 0.1520	*p* = 0.1090	*p* = 0.4820	*p* = 0.1460	*p* = 0.2700	*p* = 0.7900
Intravenous fluids (mL)	r = 0.0916	**r = −0.3273**	r = −0.1296	r = −0.2084	r = 0.0012	r = −0.0694	r = 0.0674	r = −0.0534
	*p* = 0.3930	***p*** = 0.0040 *	*p* = 0.2260	*p* = 0.0710	*p* = 0.9910	*p* = 0.5510	*p* = 0.5300	*p* = 0.6470
Urine output (mL)	r = −0.0304	r = 0.0035	r = 0.0510	**r = 0.2558**	r = 0.0613	r = −0.0132	r = −0.0282	r = −0.0735
	*p* = 0.7770	*p* = 0.9760	*p* = 0.6350	***p*** = 0.0260 *	*p* = 0.5680	*p* = 0.9100	*p* = 0.7930	*p* = 0.5280
Glasgow Outcome Scale	**r = −0.3222**	**r = 0.2566**	**r = −0.2493**	r = 0.0265	**r = 0.2770**	**r = 0.5196**	r = 0.2091	**r = 0.3369**
	***p*** = 0.0050 *	***p*** = 0.0440 *	***p*** = 0.0330 *	*p* = 0.8380	***p*** = 0.0180 *	***p*** < 0.0001 *	*p* = 0.0760	***p*** = 0.0070 *

HR = heart rate; Ht = haematocrit. * Bold type indicates statistical significance. B1—before the surgery, B2—after the induction of anaesthesia, B3—after opening the dura mater, and B4—after the surgery.

**Table 3:** Clinical outcomes in patient with preoperative hypertension (HBP) and normotension (NBP).

	HBP (n = 89)	NBP (n = 76)	*p*-Value
Glasgow Outcome Scale	2.26 ± 1.2	2.72 ± 1.2	0.0290 *
Deceased	39 (43%)	17 (22%)	0.0276 *
Length of stay (range)	12.2 ± 10.5 (1–60) days	11.8 ± 8.8 (1–37) days	0.8333

Data are presented as mean ± SD. * significantly different.

## Data Availability

The original contributions presented in the study are included in the article, further inquiries can be directed to the corresponding author.
